# Magnetoelastic Effect Detection with the Usage of Eddy Current Tomography

**DOI:** 10.3390/ma12030346

**Published:** 2019-01-22

**Authors:** Paweł Nowak

**Affiliations:** Warsaw University of Technology, Institute of Metrology and Biomedical Engineering, 02-525 Warsaw, Poland; p.nowak@mchtr.pw.edu.pl; Tel.: +48-606-421-387

**Keywords:** magnetoelastic effect, eddy current tomography, finite element method

## Abstract

The possibility of application of the eddy current tomography setup to measure the small permeability variations caused by magnetoelastic effect was presented. A ferromagnetic steel sample was prepared for applying wall stresses and measured for 30 MPa stresses. The Finite Element Method (FEM) was utilized to conduct numerical forward tomography transformation for samples of known permeability. Developed forward tomography transformation was applied for single variable inverse tomography transformation, utilized for determining magnetic permeability. This confirmed the possibility of the application of eddy current tomography for quantitative measurements of magnetoelastic effect in samples of known geometry.

## 1. Introduction

Magnetoelastic effect is a general name for several different phenomena that consider the mechanical stresses and change of a material’s magnetic properties [1]. The observable effects of those phenomena vary from change of sample dimensions due to external magnetic field (magnetostriction effect [2]) via electrical impulses due to a sample’s torsion in the magnetic field (Matteucci effect [3]) to changes in the magnetic state caused by change in the sample’s volume (Nagaoka-Honda effect [4]). One of the first reported magnetoelastic effects is the Villari effect [5]. The application of external stresses influences the ease of movement of magnetic domains in material, and thus its magnetic permeability [6]. A typical method for measuring this effect is usage of special samples for applying uniform stresses in material and measuring the magnetic hysteresis loop for different stresses values [7]. The sample may be either frame shaped [8] or ring shaped [9]. This method requires special sample preparation in order to obtain closed magnetic circuit and uniform stresses in material. Additionally, the standard method requires winding of magnetizing and sensing coils on the sample [10]. Sample winding is not required when using a contactless method such as eddy current tomography (ECT).

Eddy current tomography (or magnetic induction tomography) is a non-destructive contactless method for evaluating discontinuities in conductive materials [11]. It is based on the standard eddy current non-destructive method, where tested object influences the coupling of at least two coils. ECT setups either collects data from multiple set of coils [12] or conducts measurements for different positions of the sample [13]. Based on the measurement data, the spatial distribution of material can be obtained in a process of inverse tomography transformation [14]. 

Even most recent research considering ECT-based permeability imaging [15] had not focused on quantitative assessment of obtained values of permeability. This paper fills this gap for small permeability variations caused by the magnetoelastic effect. Additionally, the presented method is an interesting alternative for measuring of magnetoelastic effect due to its contactless nature, and thus may be suitable for stress assessment in constructions.

## 2. Materials and Methods

### 2.1. Sample Preparation

**Figure 1:**
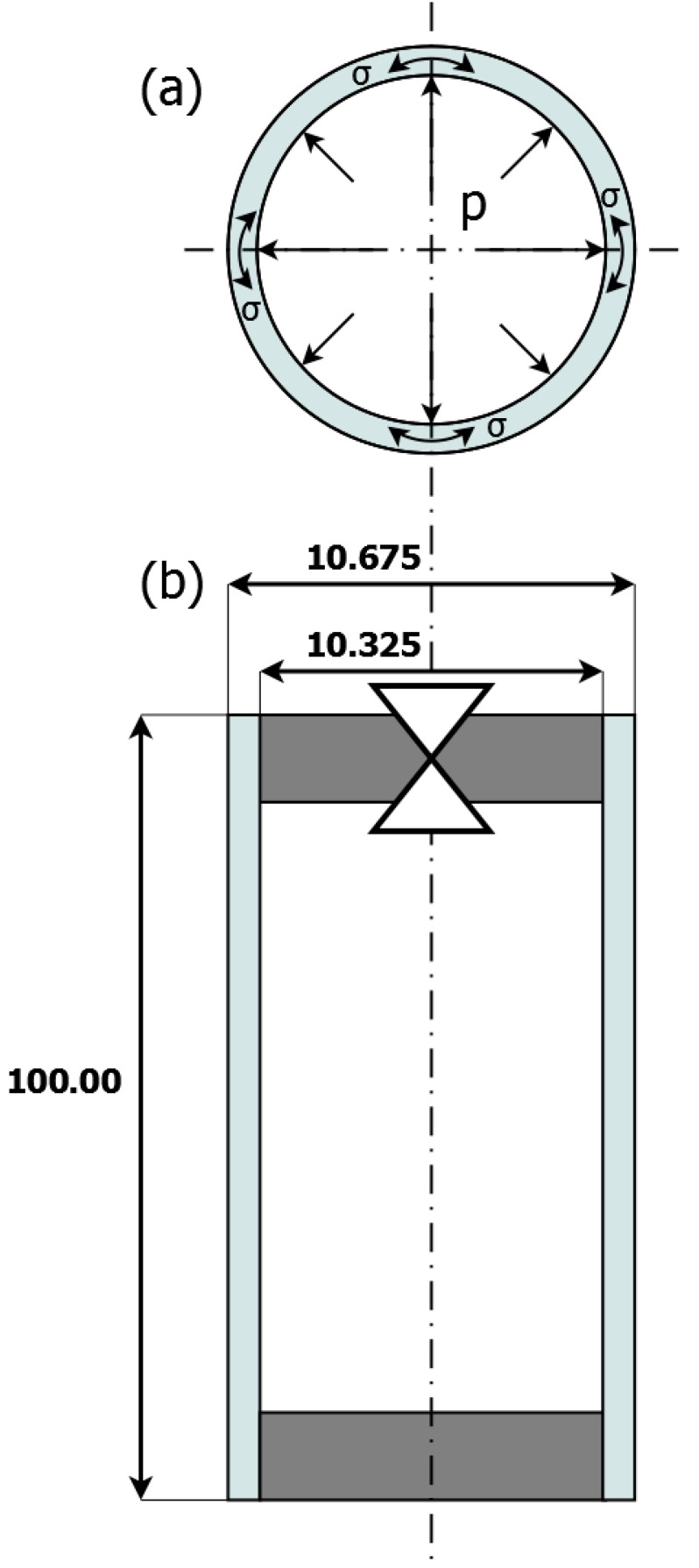
Schematic representation of the tested sample: (**a**) Cross section of the sample. Arrows indicate direction of internal pressure and stresses in material; (**b**) Dimensions of the sample with marked sealing and pressure valve.

For research, the pipe shaped sample made of 13CrMo4-5 constructional steel was considered. This material has relative magnetic permeability of 80 [16]. The length of the sample was 100.00 mm, its external radius was 10.675 mm, and wall thickness was 0.35 mm. The pipe was sealed with non-magnetic material. Additionally, in the top-side sealing was a valve to regulate the pressure inside the sample. The scheme of the sample is presented in Figure 1.

Pressure was applied into the sample through a valve with the usage of an air compressor with pressure regulation. The internal pressure caused circumferential stresses in the sample. The value of the stresses were calculated from Barlow’s formula:

(1) $$\sigma =\frac{r\cdot p}{g}$$

where *σ*—circumferential stresses in the pipe, *r*—outside radius of the pipe, *g*—thickness of the pipe wall, *p*—pressure inside the pipe.

The sample was measured without any stresses as well as with applied 1 MPa pressure, which resulted in 30 MPa circumferential stresses in the material. 

### 2.2. Measurement Method

**Figure 2:**
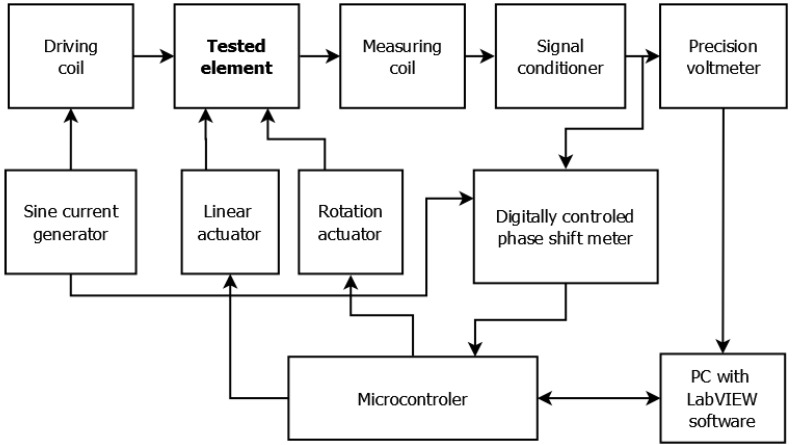
Schematic diagram of the utilized eddy current tomography setup.

**Figure 3:**
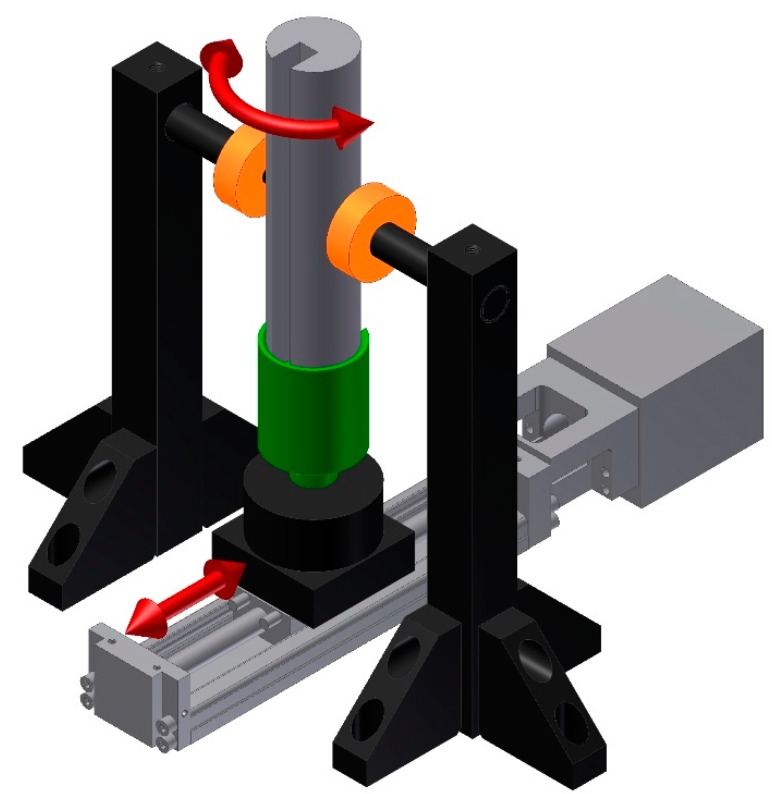
Model of eddy current tomography. Arrows indicate the movement of the sample.

The pipe was measured on an eddy current tomography setup (block diagram is presented in Figure 2., model is presented in Figure 3), described in detail in [13]. The tested sample moves linearly between two coaxial coils (driving and measuring) and, for each linear step, fully rotates around its axis in 100 discrete steps. The position of the sample is set by two stepper motors, controlled by an ARM 1114 microcontroller (NXP Semiconductors, Eindhoven, The Netherlands).

Both coils have/consist of 100 turns. The exciting coil has a 7.4 mm internal radius and 17.9 mm external radius, whereas the measuring coil has a 6.1 mm internal radius and 14.8 external radius. The driving coil is powered by a 2 kHz sine current generator and induces an alternating magnetic field, which induces eddy current in the conducting sample. The distribution of eddy current highly depends on the object’s geometry as well as on the electromagnetic parameters of the sample’s material. The magnetic field caused by the eddy current influences the magnetic field in the measuring coil. The changes concern both the amplitude of the measured field as well as phase shift between the exciting and measuring signals. The measurement of the signal’s amplitude signal is done by a 6½ digit multimeter (TH1961, Tonghui, Changzhou, China) and phase shift measurement is done by a digital phase shift meter. The entire measurement procedure is controlled by software developed in LabVIEW (National Instruments, Austin, TX, USA). 

### 2.3. Modelling Method

FEM-based forward tomography transformation was conducted with a set of open-source software. FEM modelling was done in ElmerFEM (CSC–IT Center for Science Ltd, Helsinki, Finland) [17] with the usage of a magnetodynamics solver. This software solves Maxwell’s equations in frequency domain with the usage of $\vec{A}$-V model [18].

The generation of finite element mesh was done in Netgen 5.3 (Vienna University of Technology, Vienna, Austria). For simulations, the eddy current tomography setup was reduced to 4 elements—the driving coil, measuring coil, sample model, and external ball of air. 

The driving coil was modelled as a single turn solenoid, whereas the measuring coil was modelled as a disk, in order to properly represent the phenomena of magnetic induction. In the eddy current tomography setup, measured voltage (induced due to Faraday’s law) is proportional to magnetic flux in the volume of measuring coil. In order to simplify the FEM simulations, the induced voltage was calculated as an integral of magnetic field in the volume of model of measurement coil.

The sample’s actuators were removed from the FEM model. The model of the tested sample was generated automatically in consecutive linear and angular positions. 

The fourth object in the model of eddy current tomography setup was an external air ball, which radius significantly exceeds the dimension of any other object in the model. The ball provides finite elements between the other objects in model, in order to properly simulate the distribution of the electromagnetic field. The external surface of the ball (sphere) was used for applying Dirichlet boundary conditions, in order to obtain the uniqueness of the FEM solution, which otherwise would be properly determined only up to constant.

**Figure 4:**
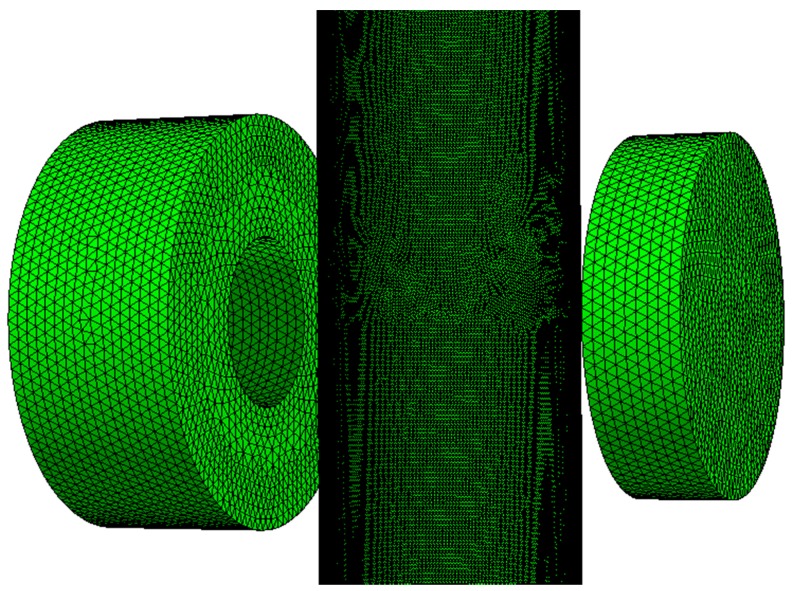
Exemplary finite element mesh utilized for FEM-based forward tomography transformation.

The solid geometry for each measurement point was automatically generated. The Netgen software, based on Delaunay algorithm, created a finite element mesh (example presented on Figure 4.). The noticeable difference of mesh density for different objects was caused by thin walls of the sample, which require a high-density mesh [19]. On average, the model of exciting coil consisted of 101,300 elements, the model of measuring coil consisted of 47,200 elements, whereas the sample’s model was formed by 755,000 1st order elements.

The forward tomography transformation is based on conducting FEM simulations for each linear and angular position of the sample. As a result of single simulations, data about distribution of magnetic field is obtained. The magnetic flux density in each finite element is described as a complex number, because utilized magnetodynamics FEM solver provides solution in the frequency domain. Magnetic flux density in the volume of measuring coil’s model is numerically integrated and data proportional to real (in phase) and imaginary (90° phase shifted) parts of inducted voltage are obtained. Thus, the values of signal amplitude (*A*) and phase shift between driving and measured signals (*P*) can be calculated based on (2) and (3):

(2) $$A=\sqrt{{\left(V_{re}\right)}^{2}+{\left(V_{im}\right)}^{2}}$$

(3) $$P=arcsin\left(\frac{V_{im}}{\sqrt{{\left(V_{re}\right)}^{2}+{\left(V_{im}\right)}^{2}}}\right)$$

where: *V_re_* and *V_im_*—integrated values of real and imaginary part of magnetic flux density in measuring coil volume.

The simulations for different measurement points can be conducted independently, which allows parallelization of the calculations. The entire procedure—generation of finite element mesh, FEM modelling and results computation—is done on a single processor core. 

### 2.4. Method of Inverse Tomography Transformation for Determining the Permeability of the Sample

**Figure 5:**
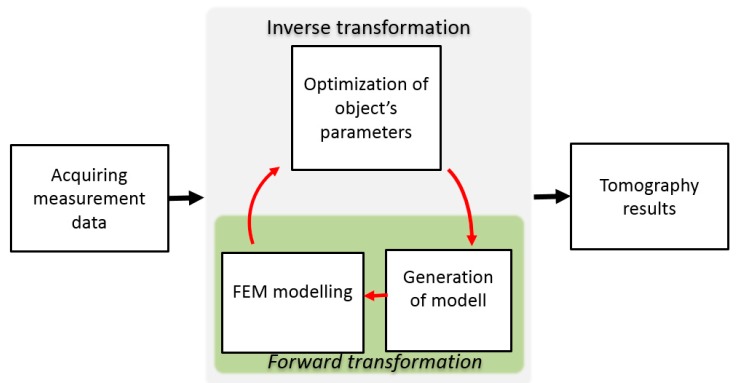
General scheme of inverse tomography transformation for eddy current tomography.

Inverse tomography transformation is used to reconstruct the properties of the measured object. Due to the fact that phenomena of eddy current induction are highly nonlinear, inverse tomography transformation requires utilization of FEM modelling as well as optimization algorithm. The objective function for the optimization algorithm is minimalization of mean difference between measurement results and FEM-based forward tomography transformation. The diagram of utilized method for inverse tomography transformation is presented in Figure 5. Initially, the measurement data are acquired. This data is compared with the normalized results of FEM-based forward tomography transformation for the given model and the value of objective function is calculated.

The object’s model may be described either by distribution of material in the cross-section of the sample [20] or by a cylindrical model with substitute defect [21]. The optimization algorithm changes the model of the sample for forward transformation in the cycle until it converges. Afterward, the tomography results are obtained as parameters of a best-fitting model.

For determining of the sample’s permeability, a downhill simplex method [22] was utilized. The sample’s geometry parameters, as well as its electrical conductivity, were set to constant. The magnetic permeability of the sample was only variable for optimization algorithm. 

## 3. Results

Due to the axial symmetry of the sample, the measurements and simulations in forward tomography transformation were conducted without rotational steps. In addition, the linear movement of the sample was limited—measurement and simulations started in initial position (45 mm from the setup’s coils axis) and were conducted with 1 mm linear step, until the sample’s midpoint reached the coil’s axis.

### 3.1. Measurement Results

**Figure 6:**
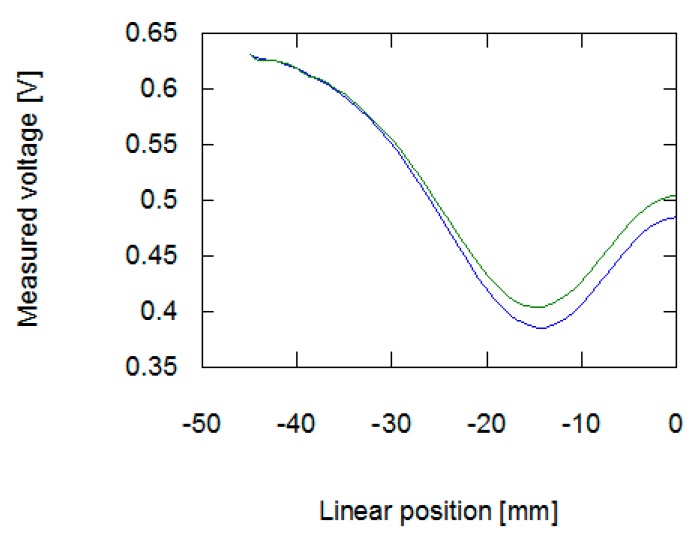
Results of measurement of amplitude of signal inducted in the measurement coil in the function of the sample’s linear position. The blue line is for a sample with no external stresses, green for a sample with 30 MPa stresses.

**Figure 7:**
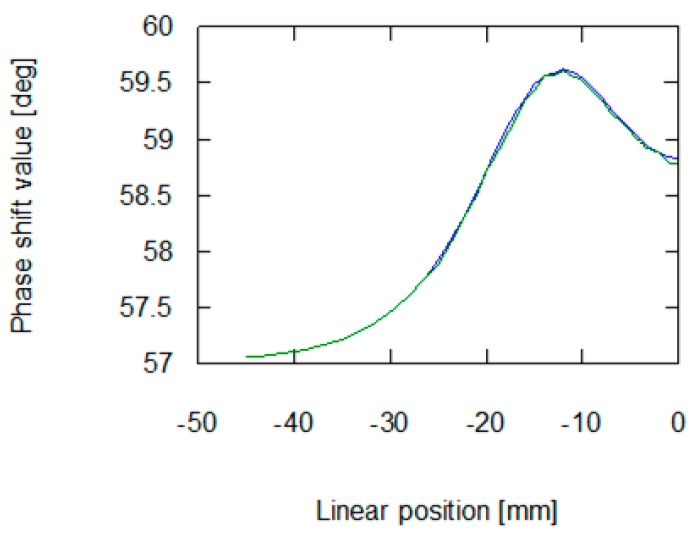
Results of measurement of phase shift between the exciting and measurement signal in the function of the sample’s linear position. The blue line is for a sample with no external stresses, green is for a sample with 30 MPa stresses.

As presented in Figure 6 and Figure 7, the application of stresses on the material noticeably influences the results of measurement on the eddy current tomography setup. The change is more noticeable in the signal amplitude measurement than in the phase shift measurement. The changes in phase shift values are more influenced by material conductivity, which was not affected by the stresses in the sample. The measurement results confirmed the ability to detect permeability changes with the eddy current tomography. Results of measurement of both signal amplitude and phase shift value exhibit the same character. Initially, the sample does not influence the magnetic field between the coils. Then, as the sample approaches the axis of the coils, the presence of conductive ferromagnetic material decreases the measured signal due to the induction of eddy currents.

### 3.2. Forward Tomography Transformation Results

**Figure 8:**
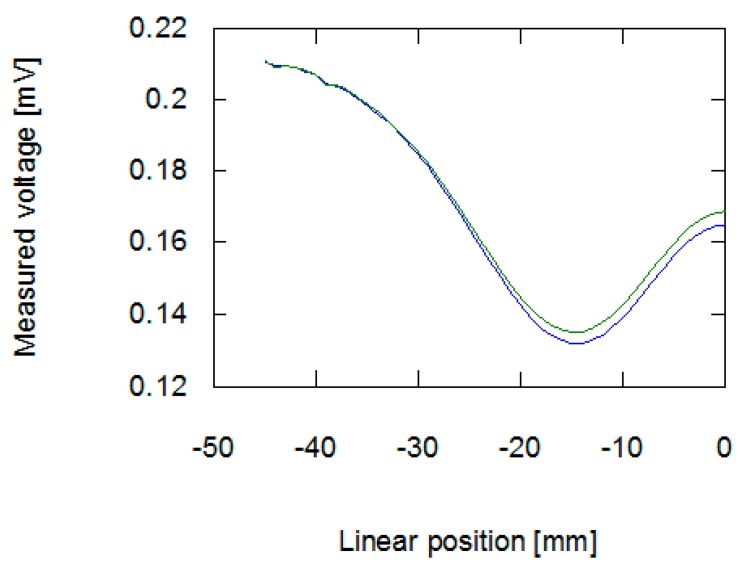
Results of forward tomography transformation values of amplitude of signal inducted in the measurement coil. The blue line is for a sample with 100 permeability, green is for a sample with 80 permeability.

**Figure 9:**
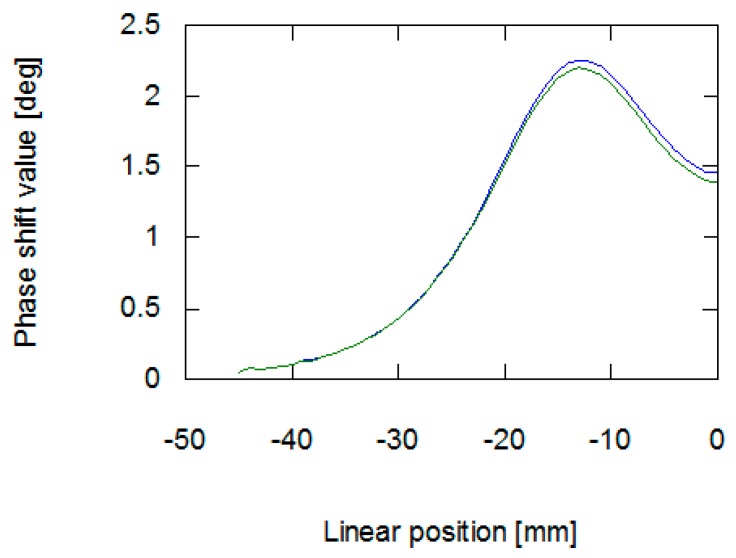
Results of forward tomography transformation values of phase shift. The blue line is for a sample with 100 permeability, green is for a sample with 80 permeability.

The results obtained from the FEM-based forward tomography are in high accordance with the measurement results. Signal amplitude and phase shift exhibit the same character of changes as presented in Section 2.2. The only difference is the scale of obtained values. The difference in the signal’s amplitudes (comparing results presented in Figure 6 and Figure 8) was caused by the usage of single-turn models of measurement and exciting coils. Additionally, in simulations, the exciting coil was supplied with a unitary current. As presented in Figure 7 and Figure 9, phase shift values obtained during the measurement have 57° offset comparing to data obtained from forward eddy current tomography transformation. The constant offset of phase shift value was caused by the signal conditioning system utilized in the tomography setup. Thus, for the purpose of proper inverse tomography transformation, the measurement and modelling data are normalized to the 0–1 range.

### 3.3. Inverse Tomography Transformation Results

As a result of inverse eddy current tomography transformation, two values of the sample’s magnetic permeability were obtained. For a sample with 0 MPa stresses, the obtained permeability value was 81.2, whereas for a sample with 30 MPa, it was 68.1. The values are in accordance with values obtained with the standard method for low magnetic fields (Rayleigh region of hysteresis loop) [12], as presented in Table 1.

## 4. Discussion

Presented in Figure 4 and Figure 5, the results of eddy current tomography measurements clearly indicate that the utilized method is suitable for distinguishing changes in material permeability caused by the Villari effect. The permeability variation notably influences signal amplitude, when the object is placed in a position near the coils (starting from 25 mm from the coils axis). On the other hand, the value of phase shift between exciting and measuring signals is not noticeably influenced. 

The results of FEM-based forward eddy current tomography present high accordance with the measurement results. The noticeable difference of range of amplitude signal changes is caused by FEM model simplifications—utilization of a unitary exciting current and a single turn coil’s models. The offset difference between the results of phase shift measurements and simulations is caused by the presence of a signal conditioner in the eddy current tomography setup. This conditioner contains a band-pass filter, which shifts the phase of the signal, as well as the amplifier, which increases the signal’s amplitude. Thus, in order to properly compare the modelling and measurement results during inverse tomography transformation, the data were normalized to the 0–1 range.

The presented method for determining sample permeability has high accordance with other methods utilized for measurements of the magnetoelastic Villari effect.

## 5. Conclusions

The possibility of qualitative analysis of magnetoelastic effect with the usage of eddy current tomography is presented. The proposed method requires the preparation of pipe-shaped samples with valves, but allows for contactless assessment of permeability changes. Additionally, inverse tomography transformation is much more time-consuming than classical methods. 

The utilized method assumed constant and known geometry of the tested sample. Further research will concern the determination of a sample’s geometry as well.

## Figures and Tables

**Table 1:** Comparison of obtained values of magnetic permeability for different sample stresses.

Sample Stresses (mpa)	M Value Obtained from Inverse Tomography Transformation	M Value Obtained with Standard Method	Relative Error (%)
0	81.2	80	1.5
30	68.1	66.8	1.9
